# UltraPlex-TMT: Expanding Isobaric Hyperplexing via Orthogonal Protease Cleavage

**DOI:** 10.1021/acs.jproteome.5c01084

**Published:** 2026-02-02

**Authors:** Theodoros I. Roumeliotis, Fernando J. Sialana, Jenny Ho, Jyoti S. Choudhary

**Affiliations:** † The Institute of Cancer Research, 5053 Chester Beatty Laboratories, London SW3 6JB, U.K.; ‡ Thermo Fisher Scientific, Stafford House, Hemel Hempstead HP2 7GE, U.K.

**Keywords:** quantitative proteomics, isobaric labeling, TMT, hyperplexing, orthogonal protease digestion, high-throughput proteomics

## Abstract

Isobaric labeling is widely used in quantitative proteomics for its multiplexing capabilities, but scaling beyond current limits remains a challenge. Here, we introduce UltraPlex-TMT, a streamlined and scalable workflow that integrates orthogonal protease digestion with hyperplex TMT/TMTpro labeling to effectively double sample throughput. UltraPlex-TMT can be readily implemented without custom chemistry or instrumentation. We benchmarked UltraPlex-TMT using lysine- and arginine-specific protease digests of a two-species proteome labeled with TMT11plex and TMT18plex across four subplexes in a proof-of-concept pseudo-58-plex design. MS2 acquisition quantified ∼6,000–7,000 proteins per subplex and ∼9,000 in total, with ∼50% overlap across all conditions, generating a robust core proteome set with high quantitative reproducibility. RTS-MS3 acquisition showed similar coverage trends, albeit with fewer quantified proteins. Despite reduced depth, MS3 data provided higher quantification accuracy, illustrating a trade-off between proteome depth and precision. Gene set enrichment analysis revealed strong biological concordance between MS2 and MS3 data, and in all conditions tested, the use of orthogonal protease digestion did not introduce systematic quantification bias. UltraPlex-TMT offers a flexible foundation for isobaric labeling-based high-throughput proteomics and is poised to benefit from faster acquisition platforms and extended multiplexing chemistries, supporting future studies exceeding 200-plex scale, potentially equivalent to subminute analysis.

## Graphical abstract

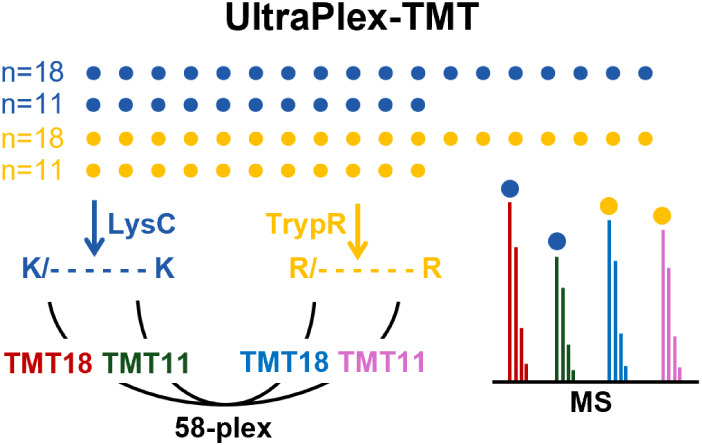

## Introduction

Isobaric labeling-based quantitative proteomics has enabled simultaneous analysis of multiple biological samples in a single experiment with high precision. Reagents such as iTRAQ^1^ and Tandem Mass Tags^2^ (TMT) have progressively increased multiplexing capabilities from 4-plex and 6-plex to 18-plex and, more recently, 35-plex formats using TMTpro reagents.^3^ These advances have enabled more cost-effective, reproducible, and scalable experimental designs across a range of biological systems. Despite this progress, the demand for higher sample throughput driven by systems biology and large-cohort studies, continues to outpace the capabilities of existing labeling chemistries. Several innovative strategies have been developed to expand the multiplexing capacity of isobaric labeling beyond the standard limits of current reagents. One of the earliest demonstrations combined triplex SILAC metabolic labeling with 6-plex TMT reagents to achieve 18-plex quantification in a single experiment.^4^ This approach layered MS1- and MS2-level information, enabling efficient comparative analysis across multiple conditions without increased instrument time. This approach has been more recently utilized to increase throughput in single-cell proteomics analysis.^5^ Similarly, the cPILOT method^6^ combines stable isotope dimethyl labeling at the peptide N-terminus with isobaric TMT labeling at lysine residues, effectively doubling the multiplexing capacity compared to TMT alone. Duplex stable isotope dimethyl labeling can also be coupled with 12-plex *N*,*N*-dimethyl leucine (DiLeu) isobaric tags to permit 27-plex quantification.^7^ Further, a hybrid TMT11 and TMTpro16 strategy, takes advantage of mass differences between the two tag sets to construct a 27-plex design.^8^ This approach can be further extended with the addition of IBT16 labeled samples to form a 45-plex assay.^9^ In an alternative approach, multiplexing capacity can be tripled by introducing Ala or Gly residues to peptides prior to TMTpro labeling with additional reaction steps.^10^ With the incorporation of IBT16 labeling the latter approach can form a 102-plex assay.^11^

Inspired by the empirical observation that consistent protein-level quantification can be achieved across different TMT-labeled data sets, even when distinct peptides from the same protein are used, we hypothesized that hyperplexing could also be accomplished by combining parallel protease digests. Specifically, we reasoned that two sets of samples digested with different proteases but labeled with the same isobaric reagent set (e.g., TMTpro-18plex), could be mixed and analyzed together in a single experiment. Due to the orthogonal cleavage specificities of the proteases, each set generates nonoverlapping peptide precursor populations. This difference enables confident discrimination between the two subplexes during database search, using appropriate enzyme-specific parameters. Crucially, this approach is compatible with existing hyperplexing methods, further expanding their sample capacity to enable UltraPlex-TMT designs without introducing new chemistries, tags, or acquisition modes.

In this study, we demonstrate the utility of an UltraPlex-TMT design that enables 58-plex pseudomultiplexed analysis by combining TMT and TMTpro isobaric labeling with orthogonal lysine- and arginine-specific protease digestion. We benchmark its performance using a two-species proteome system to assess proteome coverage, quantification reproducibility, and accuracy highlighting its potential for large-scale, high-throughput proteomics.

## Methods

### Cell Lines

For the colorectal cancer cell lines, frozen cell pellets collected during a previous study from our group were used and are described in Roumeliotis et al.^12^ Briefly, cells were grown in either a DMEM/F12 medium (Gibco) supplemented with 10% fetal calf serum (v/v) (Gibco) and 50 U/mL penicillin and 50 mg/mL streptavidin (Gibco), or an RPMI 1640 medium (Gibco) supplemented with 10% fetal calf serum (v/v) (Gibco), 50 U/mL penicillin, 50 mg/mL streptavidin (Gibco), 2.5 mg/mL glucose (Sigma), and 1 mM sodium pyruvate (Gibco), and maintained at 37 °C in a humidified atmosphere at 5% CO2. The cells were harvested by incubation with TrypLE (Gibco) until detached and washed twice with a cold DPBS solution before snap freezing on dry ice. Each pellet contained approximately 3 × 10^6 cells.

### Sample Preparation for Pseudo-58-plex Analysis

Sample preparation was conducted using an adaptation of our SimPLIT workflow.^13^ Four cell pellets corresponding to four different colorectal cancer cell lines were lysed in a buffer containing 1% sodium deoxycholate (SDC, Merck, Cat. No. 30970), 100 mM triethylammonium bicarbonate (TEAB, Merck, Cat. No. T7408), 10% isopropanol and 50 mM NaCl, freshly supplemented with 5 mM TCEP (Thermo Fisher Scientific, Bond-Breaker, Cat. No. 77720), 10 mM iodoacetamide (IAA, Merck, Cat. No. I1149), universal nuclease 1:2000 vol/vol (Pierce, Cat. No. 88700) and Halt protease and phosphatase inhibitor cocktail (Thermo Fisher Scientific, Cat. No. 78442, 100X) with 5 min of bath sonication. Protein concentration was measured with the Quick Start Bradford protein assay (Bio-Rad, Cat. No. 5000205). Aliquots of 30 μg of total protein from each cell line were transferred in a 96-well plate according to the 58-plex design shown in Figure 1C. An *E. coli* protein extract (Bio-Rad, Cat. No. 1632110) was added at 1 μg or 0.5 μg across the human cell lysates as shown in Figure 1C. The plate was SpeedVac dried and the samples for the arginine-specific cleavage set (TrypR) were reconstituted in 25 μL (TMT11 subset) or 12.5 μL (TMT18 subset) of 100 mM TEAB buffer followed by the addition of 10 μL TMT11 (stock 20 μg/μL, Thermo Fisher Scientific, Cat. No. A34808) or 5 μL TMTpro18 (stock 25 μg/μL, Thermo Fisher Scientific, Cat. No. A52045), according to the design in Figure 1C, and 1h reaction at room temperature for protein-level TMT labeling. The TMT labeled samples were SpeedVac dried, and all samples (labeled and unlabeled) were reconstituted in 20 μL of 100 mM TEAB buffer. Proteins were digested overnight at room temperature by the addition of 2 μL LysC (Thermo Fisher Scientific, Cat. No. 90307) or Trypsin (Thermo Fisher Scientific, Cat. No. 90059) stock solution of 500 ng/μL in 0.1% formic acid (final enzyme 50 ng/μL, 1:30) for the LysC and TrypR sets, respectively. The TrypR peptides were then fully labeled by another addition of 10 μL TMT11 or 5 μL TMTpro18. The LysC peptides were labeled by the addition of 10 μL TMT11 or 10 μL TMTpro18. The reaction was quenched after 1h with the addition of 2 μL 5% hydroxylamine solution and premix samples were prepared by pooling 0.5 μL from each sample into 100 μL of 0.1% TFA for each subplex separately. In each premix, SDC was precipitated with addition of formic acid at 2% and centrifugation at 20,000 × *g* for 5 min. Supernatants were transferred into clean vials for LC-MS analysis. Following evaluation of the premix runs, samples were pooled into four subplexes, diluted with 100 μL of HPLC water, heated at 95 °C for 5 min, cooled down and heated for another 5 min. Lastly, each pool was acidified with formic acid at 2%, the precipitated SDC was removed by centrifugation and supernatants were SpeedVac dried.

**Figure 1:**
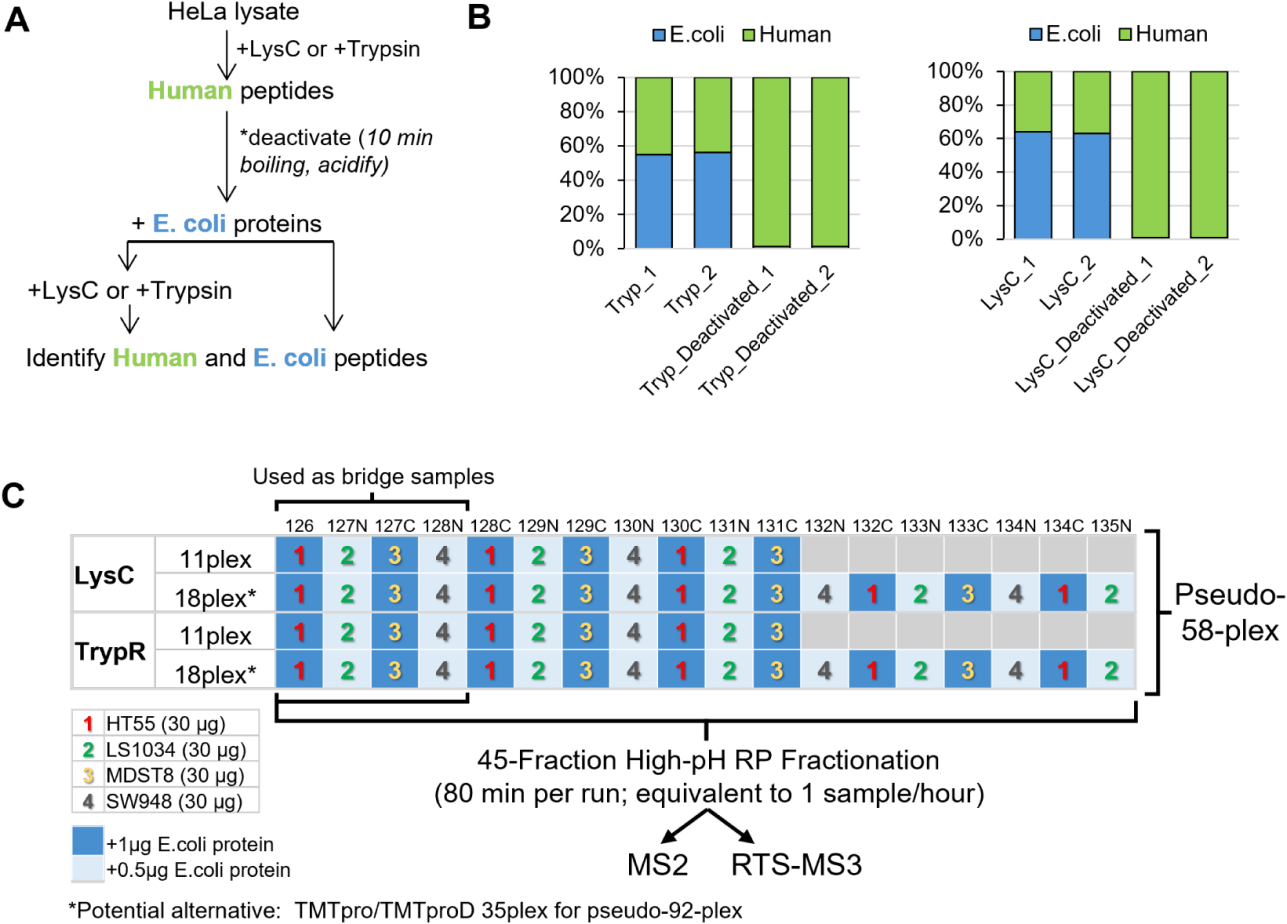
Protease deactivation evaluation and pseudo-58-plex study design. (A) Experimental workflow for Trypsin and LysC deactivation evaluation. (B) Stacked bar plots showing the percentage of Human and *E. coli* peptides per enzyme under active or deactivated status. (C) Design matrix for the UltraPlex-TMT pseudo-58-plex experiment. Colored numbers correspond to four different colorectal cancer cell lines; blue and light blue cells indicate spike-in *E. coli* amounts of 1 and 0.5 μg, respectively. Gray cells represent unused TMT channels. The experiment is comprised of four subplexes: LysC-TMT11, LysC-TMT18, TrypR-TMT11, TrypR-TMT18. Two data sets were collected from the same samples using MS2 and Real-Time-Search (RTS)-MS3 acquisition.

### Sample Preparation for Enzyme Deactivation Evaluation

Approximately two million HeLa cells (Ipracell, batch CC011010) were lysed as described above. Following Bradford assay, aliquots containing 30 μg of protein in 18 μL were digested with either Trypsin or Lys-C at a 1:10 and 1:30 enzyme-to-protein ratio, respectively. Samples were incubated for 2 h at 37 °C, followed by overnight incubation at room temperature. To quench residual protease activity, digested samples were boiled for two cycles of 5 min at 95 °C. After thermal treatment, samples were acidified with 2 μL of formic acid, diluted to 100 μL with water, centrifuged at 20,000 × *g* for 5 minutes to remove precipitated SDC, and SpeedVac dried. Dried peptides were reconstituted in 100 μL of LC-MS-grade water, sonicated in water bath, and split into two aliquots of 50 μL each to generate matched control and deactivated groups. Control samples were supplemented with 50 μL of 200 mM TEAB, while deactivated samples received 50 μL of 0.2% ammonium hydroxide. All samples were spiked with 7.5 μL of 2 μg/μL *E. coli* protein lysate (Bio-Rad, Cat. No. 1632110) before secondary digestion to assess residual protease activity. Additional proteases were added only to control samples: 3 μL of 0.5 μg/μL Trypsin or 1 μL of 0.5 μg/μL Lys-C. No additional enzymes were added to the deactivated samples. All samples were incubated overnight at room temperature to allow digestion. Peptides were then acidified to precipitate residual SDC and centrifuged. Supernatants were desalted using C18 tips, SpeedVac dried, and reconstituted in 0.1% formic acid before LC-MS analysis.

### High-pH Reversed-Phase Peptide Fractionation

All four subplexes were pooled in 100 μL of 0.1% (v/v) ammonium hydroxide and the final peptide pool was fractionated with high pH reversed-phase chromatography using the XBridge C18 column (2.1 × 150 mm, 3.5 μm, Waters) on an UltiMate 3000 HPLC system over a 1% gradient in 35 min. Mobile phase A was 0.1% (v/v) ammonium hydroxide and mobile phase B was 0.1% ammonium hydroxide (v/v) in acetonitrile. Fractions were collected every 30 s and eventually pooled into 45 samples for LC-MS analysis.

### LC-MS Analysis

LC-MS analysis for the UltraPlex-TMT experiment was performed on a Vanquish Neo UHPLC (Thermo Fisher Scientific) system coupled to the Orbitrap Ascend mass spectrometer (Thermo Fisher Scientific) using a 25 cm capillary column (nanoE MZ PST BEH130 C18, 1.7 μm, 75 μm × 250 mm, Waters Cat. No. 186008795) over a 70 min gradient 5%–35% of mobile phase B composed of 80% acetonitrile, 0.1% formic acid. Peptides were dissolved in 0.1% TFA and approximately 3 μg were loaded and preconcentrated onto a PepMap 100, C18, 5 μm, 0.3 × 5 mm, 1500 bar, (Thermo Scientific Cat. No. 174500) trapping column following elution in the analytical column which was attached to a Nanospray Flex ion source via a stainless-steel emitter (Thermo Scientific, Cat. No. ES542). For both MS2 and RTS-MS3 acquisition methods, MS1 spectra were collected at Orbitrap mass resolution of 120,000. For the MS2 method, multiply charged precursors were selected for HCD fragmentation in the top speed mode (3 s) with collision energy 38% and Orbitrap detection at a resolution of 45,000 with 91 ms maximum injection time. For the RTS-MS3 acquisition, multiply charged precursors were selected for MS2 HCD fragmentation with collision energy 32% with iontrap detection in turbo scan rate (35 ms maximum injection time). MS3 scans were triggered by Real Time Search (RTS) against a FASTA file containing UniProt *Homo sapiens* reviewed canonical sequences concatenated with *Escherichia coli* entries using synchronous precursor selection (SPS) (up to 10 notches) and MS3 HCD fragmentation with collision energy 65% at 45,000 Orbitrap resolution. Carbamidomethyl at C (+57.0215) was selected as static modification and TMTpro16plex at K/n-term (+304.2071), TMT6plex at K/n-term (+229.1629), Deamidation of N/Q (+0.984) and Oxidation of M (+15.9949) were selected as variable modifications with maximum 2 trypsin missed-cleavages and 2 variable modifications per peptide. Precursors were dynamically excluded from further activation for 45 s with 10 ppm mass tolerance. The LC-MS analysis for the premix runs was performed with an 80 min gradient on the same platform, with MS2 acquisition using HCD fragmentation with collision energy 32% at 45,000 Orbitrap resolution. The LC-MS analysis for the enzyme deactivation experiments were performed on an UltiMate 3000 UHPLC coupled to the Orbitrap Lumos mass spectrometer (Thermo Fisher Scientific) over a 120 min gradient 5%–40% of mobile phase B (80% acetonitrile, 0.1% formic acid) with MS2 acquisition using HCD fragmentation with collision energy 32% and iontrap detection.

### Data Processing

The Sequest HT and Comet nodes in Proteome Discoverer 3.0 (Thermo Fisher Scientific) were used to search the raw files of the UltraPlex-TMT experiment against FASTA files containing reviewed UniProt *Homo sapiens* and *Escherichia coli* entries. The precursor mass tolerance was set at 20 ppm and the fragment ion mass tolerances were 0.02 Da for the MS2 and 0.5 (Sequest HT) or 1.0 Da (Comet) for the MS3 data, with up to 2 missed-cleavages allowed for each enzyme. The raw files were processed four times, corresponding to the four distinct subplexes. In all searches, variable modifications included oxidation (+15.995 Da) on methionine (M) and deamidation (+0.984 Da) on asparagine (N) and glutamine (Q). Carbamidomethylation (+57.021 Da) on cysteine (C) was set as a static modification. For TMT11 and TMTpro18 subplexes, TMT6plex (+229.163 Da) and TMTpro (+304.207 Da) modifications, respectively, were set as static on peptide N-termini and lysine (K) residues. LysC and Trypsin (R, P-inhibitor) were selected as the proteases in the respective subplexes. Peptide confidence was estimated with the Percolator node and peptide FDR was set at 0.01 based on target-decoy search. Only unique peptides were used for MS2 or MS3 quantification, considering protein groups for peptide uniqueness. Peptides with average reporter signal-to-noise greater than 3 were used for protein quantification, and correction for isotopic impurities was applied. The raw files from the individual subplex premix runs were processed as described above for the MS2 data, using only the Sequest HT search engine once with the Trypsin (KR, P-inhibitor) and once with the LysC or Trypsin (R, P-inhibitor) setting according to the respective subplexes, to assess protease specificity and missed-cleavage rates. Carbamidomethylation (+57.021 Da) of cysteine (C) was set as a dynamic modification to assess alkylation efficiency. For the enzyme deactivation experiments, Sequest HT search settings specified Trypsin or LysC as the proteolytic enzyme according to the respective samples, with a fragment ion mass tolerance of 0.5 Da.

### Statistical Analysis

Data normalization and scaling was performed with an in-house script in RStudio. For the human proteins, raw protein-level signal-to-noise values were exported from Proteome Discoverer 3.0, and only Master proteins with at least one nonzero value across samples within a subplex were retained for further analysis. Zero values were replaced with a minimum value of 0.1 to enable ratio generation, followed by sample-wise median normalization and log2 transformation. The log2-transformed data were further scaled by subtraction of the mean of the four bridge channels per subplex, and data were finally centered at zero across all samples from the different subplexes using only proteins quantified across all subplexes. For the *E. coli* proteins, log2 ratios were directly computed from the raw protein-level signal-to-noise values. Principal variance component analysis (PVCA) was performed in the BatchServer^14^ software. Gene set enrichment analysis (GSEA) was performed in the Perseus platform^15^ by applying 1D annotation enrichment^16^ on the median log2 scaled values per cell line against Hallmark gene sets. Significant GSEA terms, boxplots and density plots were visualized in RStudio with the ggplot2 package. Heatmaps and correlation matrices were plotted in Phantasus.^17^ To harmonize the MS2 and MS3 data sets, log2-relative ratios were normalized by dividing each column by its median absolute deviation (MAD), thereby adjusting for differences in dynamic range across acquisition methods.

## Results

### Orthogonal Cleavage Strategy

To enable orthogonal proteolysis suitable for hyperplexing, we devised a strategy to generate nonoverlapping peptide populations at the MS1 level by performing parallel enzymatic digestion. Proteins are digested either with LysC, which cleaves C-terminal to lysine residues, or with Trypsin after lysine residues have been fully blocked by TMT labeling. As TMT modification prevents cleavage at lysine, Trypsin activity is restricted to arginine sites (hereafter referred to as TrypR), similarly to previous methods for characterization of histone modifications.^18^ This approach yields two distinct subplexes: one derived from LysC cleavage and the other from Arg-specific Trypsin cleavage. We excluded the use of the common Arg-C enzyme, as it exhibits off-target activity at lysine residues, which could compromise the orthogonality of the cleavage strategy by generating overlapping peptides with LysC. The use of ArgC Ultra could provide a highly specific arginine cleavage option to further simplify the workflow; however, this reagent was not available at the time the experiments in this study were performed.^19^ Because hyperplexing ultimately requires pooling of differently digested samples, complete inactivation of enzymatic activity prior to mixing is essential to avoid cross-digestion. To evaluate the efficiency of enzyme deactivation, we performed a control experiment using HeLa cell extracts digested with either LysC or Trypsin, followed by sequential boiling (10 min) and acidification (Figure 1A). After this inactivation step, an *E. coli* protein extract was added to each sample. The samples were then split: one-half received fresh enzyme as a positive control, while the other half was left untreated to assess residual activity. To simulate downstream workflow conditions, the second incubation step was carried out in 0.1% (v/v) ammonium hydroxide, corresponding to high-pH mobile phase A used in offline fractionation. LC-MS analysis followed by database searching against human and *E. coli* proteomes showed that, on average, only ∼1% of peptides in the untreated samples were derived from *E. coli*, whereas in the positive controls, comparable levels of *E. coli* and human peptides were detected (Figure 1B). These results confirm that the combined boiling and acidification protocol is effective in inactivating LysC and Trypsin and is compatible with the subsequent pooling and high-pH fractionation steps in the hyperplexing workflow.

### UltraPlex-TMT Design for Pseudo-58-plex Analysis

To benchmark the performance of our orthogonal proteolysis and hyperplexing strategy, we implemented a pseudo-58-plex design by combining four subplexes generated from distinct combinations of enzyme-specific cleavage and TMT labeling reagents. Specifically, two sets of digests, each comprising 29 samples, were prepared using either LysC or TrypR digestion. Within each digestion set, samples were labeled with either TMT 11-plex or TMTpro 18-plex reagents, resulting in 29 reporter channels per digestion type and a total of 58 channels across the experiment (Figure 1C). The four resulting subplexes are hereafter referred to as LysC-TMT11, LysC-TMT18, TrypR-TMT11, and TrypR-TMT18. To enable quantitative integration across all samples, identical bridge samples were assigned to the same reporter channels (TMT tags 126–128N) in all subplexes, providing a common reference for batch effect correction.

Each TMT channel contained 30 μg of protein derived from one of four colorectal cancer cell lines: HT55, LS1034, MDST8, and SW948 that we previously characterized using proteomics and phosphoproteomics.^12^ These were distributed across the labeling channels in a randomized but balanced fashion to test quantification reproducibility and proteome depth. Additionally, an *E. coli* protein lysate was spiked into all samples at 1 μg or 0.5 μg to designated TMT channels to assess quantification accuracy. Sample preparation was performed on a 96-well plate using an adaptation of our one-pot SimPLIT workflow.^13^

The final pooled sample, combining both LysC and TrypR digests, represents a pseudo-58-plex configuration (29 channels × 2 digestion types), offering an expanded quantitative capacity compared to conventional single-enzyme TMT designs. The pooled sample was then subjected to high-pH reversed-phase fractionation using a 45-fraction scheme (80 min per fraction), currently equivalent to ∼1 sample per hour of MS acquisition time. All fractions were analyzed once with an MS2 and once with a Real-Time-Search (RTS) MS3 LC-MS method. The raw data were processed four times for protein identification and quantification using the appropriate settings per enzyme and TMT type combination. It is expected that the combination of the four subplexes will result in increased MS1 complexity, with implications for proteome coverage and quantitative accuracy due to coisolation interference. Therefore, the extended fractionation in this workflow was selected to maximize coverage and quantification depth while maintaining efficient throughput and accuracy for the pseudoplex experiment. As an alternative future design, we propose replacing the 18-plex with TMTpro–TMTproD 35-plex reagents, which would enable a pseudo-92-plex experiment when paired with the same orthogonal cleavage and bridge normalization strategy.

### Evaluation of Digestion and Labeling Efficiency Prior to UltraPlex-TMT Pooling

To assess digestion, enzyme specificities and TMT labeling efficiencies prior to pooling the full 58-plex experiment, we performed preliminary quality control runs on small pools using aliquots from each sample within the LysC and TrypR subplexes. For each digestion set, LysC-TMT11, LysC-TMT18, TrypR-TMT11, and TrypR-TMT18, equal amounts of peptide material were combined into a premix and analyzed in single-shot LC-MS mode. To confirm the specificity of the orthogonal digestion strategy, database searches were performed for each TMT-labeled premix using trypsin with K and R cleavage as the search setting. For the LysC digests, ∼88% of peptides showed lysine-specific cleavage, with ∼11% containing a single arginine cleavage site (, left panel). Importantly, peptides containing two arginine cleavages, potentially interfering with the TrypR digest, were detected at <0.4%. For the TrypR digests, ∼96% of peptides were cleaved at arginine, while only ∼1% contained two lysine cleavages, a potential source of interference with LysC digests (, right panel). These results demonstrate that both proteases retained high specificity, with any nonspecific cleavage confined to acceptable levels. Next, we repeated the database searches in the premixes using the matched enzyme settings to assess the missed cleavage rates. As shown in , the percentage of peptide-spectrum matches (PSMs) with zero missed cleavages was over 97% across all subplexes, indicating robust proteolytic activity and supporting the fidelity of the orthogonal digestion strategy. Additionally, summarizes TMT N-terminal and lysine labeling efficiency, which was uniformly high across all conditions, ranging from 99.43% to 99.97%, confirming effective labeling and minimal variability between TMT reagent batches. These results validated the enzymatic digestion specificity and labeling consistency across subplexes, justifying downstream pooling and offline fractionation for the full 58-plex design.

### Evaluation of Peptide Metrics across Subplexes

Following full sample pooling, high-pH reversed-phase fractionation, and LC-MS analysis, we evaluated multiple peptide- and spectrum match-level metrics to assess the quantitative and qualitative performance of each UltraPlex-TMT subplex. As shown in , the majority of peptide-spectrum matches (PSMs) in the MS2 data set were assigned to charge states +2 and +3 across all conditions, with a notable proportion of +4 species also observed; likely reflecting increased peptide lengths due to the lower frequency of cleavage sites when using LysC or TrypR individually. Notably, both TMT18-labeled subplexes displayed an increase in triply charged precursors compared to their TMT11plex counterparts for both LysC and TrypR digestions. This shift is likely attributable to the increased mass and altered physicochemical properties of the TMTpro reagents used in the 18-plex conditions. The median precursor ion intensity was approximately 17% higher in TMT18plex compared to TMT11plex, likely reflecting the increased number of samples (18 vs 11) contributing to each pool while maintaining equal protein input per channel (). The median reporter ion signal-to-noise ratio remained high (>170) and consistent across all conditions (). Overall, the MS2 acquisition yielded a slightly higher number of identified peptide groups than RTS-MS3 and, as expected, LysC digests consistently yielded more peptide groups than TrypR, likely due to more favorable peptide length distributions. TMT18 labeling resulted in a higher number of identified peptide groups than TMT11 for both LysC and TrypR digestions (). While this may appear counterintuitive, given that previous studies have reported approximately 10% fewer peptide identifications when using TMTpro reagents compared to TMT10plex,^20^ the observed increase is likely attributable to the higher total peptide load in the TMT18 subplexes, stemming from the inclusion of more labeled samples per pool. Together, these data align with expectations given the UltraPlex-TMT design, with TMT18 benefiting from higher peptide load and LysC digestion yielding greater peptide coverage than TrypR due to its more frequent cleavage pattern.

### Quantitative Proteome Coverage

To evaluate performance at the protein level across the UltraPlex-TMT design, we assessed the number of proteins quantified in each subplex using both MS2-based and real-time search MS3 (RTS-MS3) acquisition strategies. As shown in Figure 2A, all subplexes quantified between 6,000 and 7,000 proteins using MS2 acquisition, with a slightly higher number detected in the TMT18-labeled samples (). Notably, a consistent number of *E. coli* proteins were also quantified across conditions, confirming reproducible detection of the internal spike-in control. A total of 9,122 unique proteins were quantified in at least one of the MS2 subplexes (Figure 2B), with nearly 49% (4,429 proteins) shared across all four conditions, reflecting a substantial core proteome. Notably, 5,550 proteins were quantified in common between the TrypR-TMT18 and LysC-TMT18 subplexes, demonstrating that using orthogonal protease digestion as an alternative hyperplexing strategy, rather than for ultraplexing, can further enhance proteome depth at the cost of reduced sample throughput. The RTS-MS3 acquisition results (Figure 2C) followed similar trends, albeit with a reduced number of quantified proteins, ranging between 5,000 and 6,000 per subplex. The total number of proteins quantified using RTS-MS3 across all subplexes was 8,000 (Figure 2D), with 41% (3,268 proteins) shared by all conditions. This reduction in the number of quantified proteins in RTS-MS3 was notably larger than anticipated based on the relative number of identified peptides between MS2 and RTS-MS3. This discrepancy can be attributed to differences in peptide identification between the postacquisition database search and the real-time search (RTS) engine, which may have failed to trigger MS3 scans for certain peptides despite their presence in the data set. Together, these results confirm the robustness and depth of the UltraPlex-TMT design across acquisition strategies. They also highlight the potential trade-offs between proteome coverage and sample throughput when optimizing multiplexed workflows.

**Figure 2:**
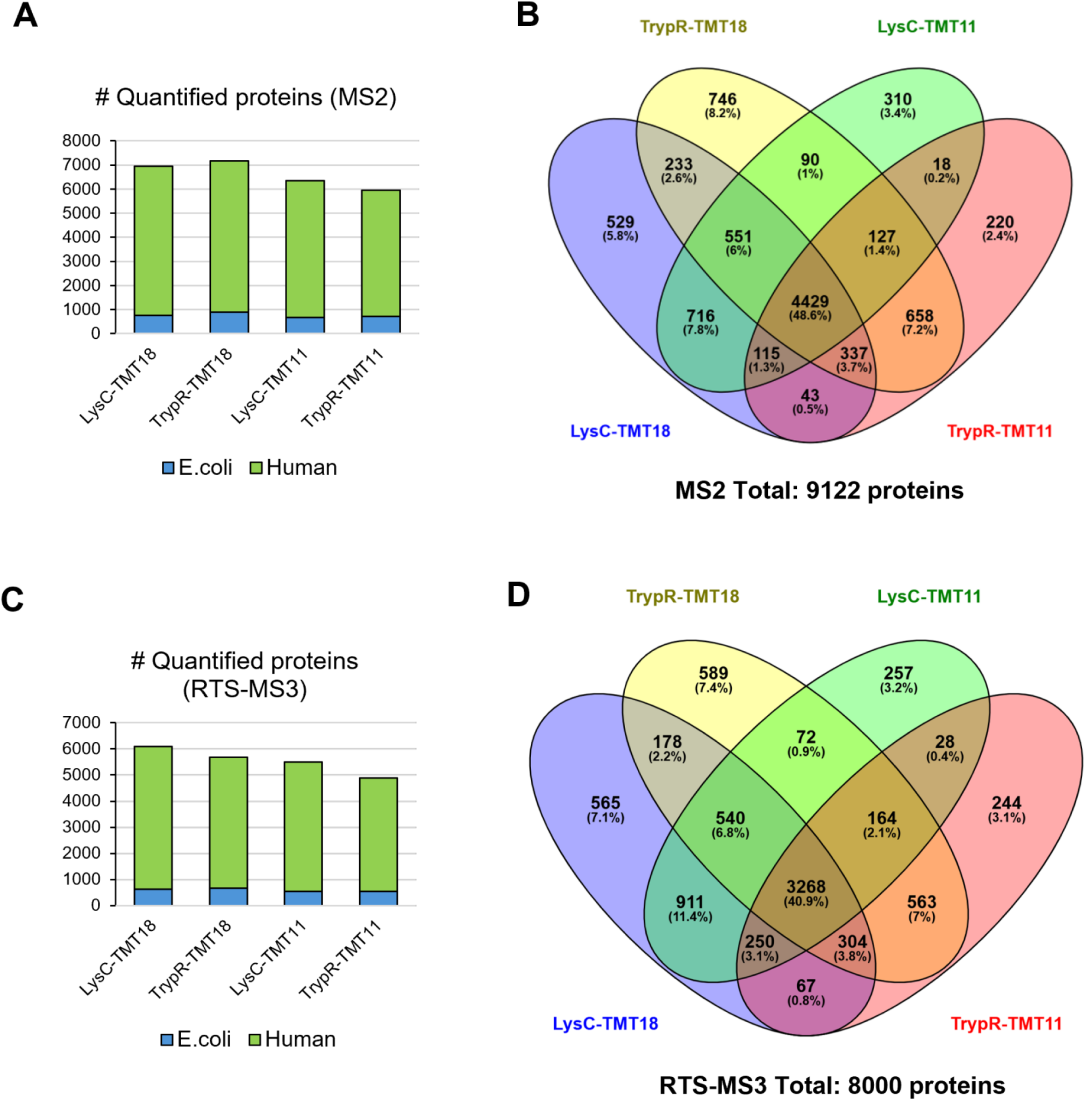
Proteome coverage and overlap across UltraPlex-TMT subplexes. (A and C) Stacked bar plots showing number of quantified human and *E. coli* proteins in each of the four UltraPlex-TMT subplexes using MS2 (A) and RTS-MS3 (C) acquisition methods. (B and D) Venn diagrams illustrating overlap in quantified proteins across the four UltraPlex-TMT subplexes for MS2 (B) and RTS-MS3 (D) data sets.

### Quantitative Consistency across Subplexes

To assess the reproducibility of the UltraPlex-TMT workflow, we compared the protein quantification profiles of the four colorectal cancer cell lines across all TMT subplexes. Hierarchical clustering of log2-transformed relative ratios from both MS2 and RTS-MS3 acquisitions (Figure 3A) showed strong grouping by cell line, indicating that biological variability outweighed technical differences across TMT types and digestion enzymes. Principal variance component analysis (PVCA, Figure 3B) quantitatively confirmed this: cell line identity accounted for over 99% of the total variance in both the MS2 and RTS-MS3 data sets, with negligible contributions from enzyme, TMT type, or acquisition strategy. Additionally, the median coefficient of variation (CV) at the protein level remained largely below 10% across replicates, both within and between the label and enzyme subplexes. As expected in multibatch designs, intersub-plex CVs were slightly elevated, but still within acceptable limits for quantitative consistency ().

**Figure 3:**
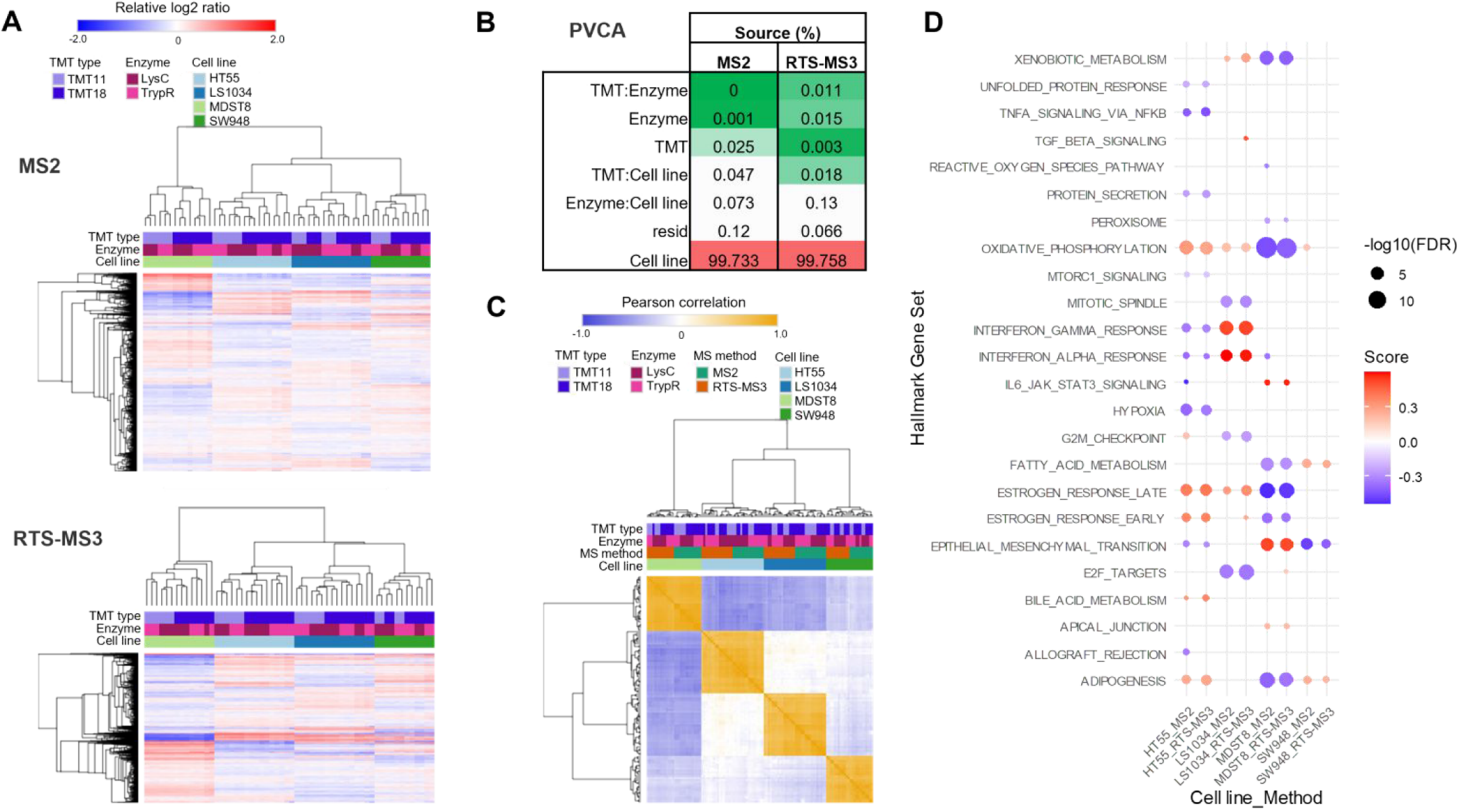
Reproducibility of UltraPlex-TMT quantification within and across acquisition modes and biological consistency. (A) Hierarchical clustering of protein expression profiles across all cell lines, TMT types, and enzymes using MS2 (top panel) and RTS-MS3 (bottom panel) acquisition. (B) Principal variance component analysis (PVCA) of the MS2 and RTS-MS3 data sets showing the proportion of total variance attributable to each factor or interaction. Residual variance component is noted as “resid”. (C) Pairwise correlation heatmap of all samples grouped by cell line, acquisition method, protease, and TMT type. (D) Dot plot of gene set enrichment analysis (GSEA) results comparing Hallmark gene sets enriched in MS2 and RTS-MS3 data sets per cell line.

A hierarchical clustering heatmap using proteins quantified in both data sets highlighted a noticeable difference in dynamic range between MS2 and RTS-MS3 acquisition methods, due to reporter ion interference and compression in MS2 (). To mitigate this discrepancy and enable more comparable downstream analyses, we normalized the log2 ratio data by dividing each column by its median absolute deviation (MAD), as shown in , lower heatmap. This adjustment effectively scales the data to account for method-specific dynamic range differences while preserving relative expression patterns. This normalization enhanced cross-platform comparability and confirmed high correlation across replicate conditions, again dominated by cell line identity rather than technical variables (Figure 3C).

Despite the known reporter ion ratio compression associated with MS2 acquisition, gene set enrichment analysis (GSEA) based on the intersection of commonly quantified proteins revealed highly concordant biological signatures between MS2 and RTS-MS3 data sets (Figure 3D). This demonstrates that, even with reduced quantitative accuracy, MS2 data can yield comparable biological interpretations to RTS-MS3, reinforcing its utility in large-scale comparative proteomics.

### Evaluation of Quantification Accuracy Using *E. coli* Spike-in Proteins

To assess quantification accuracy across acquisition methods and subplex designs, we examined the log2 ratios of *E. coli* spike-in proteins, which were mixed at a known 2:1 ratio (expected log2 ratio = 1). In MS2-based acquisitions, the median *E. coli* log2 ratio across all samples was 0.57 (Figure 4), indicating ratio compression, consistent with known coisolation interference effects in MS2 workflows. Interestingly, the TMT18-labeled subplexes showed slightly higher median log2 ratios than their TMT11 counterparts. This difference was attributable to the inclusion of unique reporter channels in TMT18, specifically 132N–135N, that do not overlap with the standard TMT11 set and therefore likely experience reduced precursor interference. This pattern was clearly reflected in the channel-level boxplots of *E. coli* protein abundances (), where these TMT18-specific channels consistently exhibited ratios closer to the expected value. In contrast, RTS-MS3 acquisition, which uses real-time ion selection to minimize interference, resulted in significantly improved quantification accuracy overall, with a median *E. coli* log2 ratio of 0.88 (Figure 4). Across all subplexes, the spread and central tendency of *E. coli* ratios were more consistent in RTS-MS3, regardless of TMT type or enzyme used, confirming the benefit of MS3-level interference correction for accurate reporter ion quantification. Together, these results highlight how both acquisition strategy and reporter channel selection affect quantification accuracy in hyper-plexed proteomics, and that the use of orthogonal protease digestion does not introduce systematic quantification bias, supporting its suitability for advanced multiplexing strategies.

**Figure 4:**
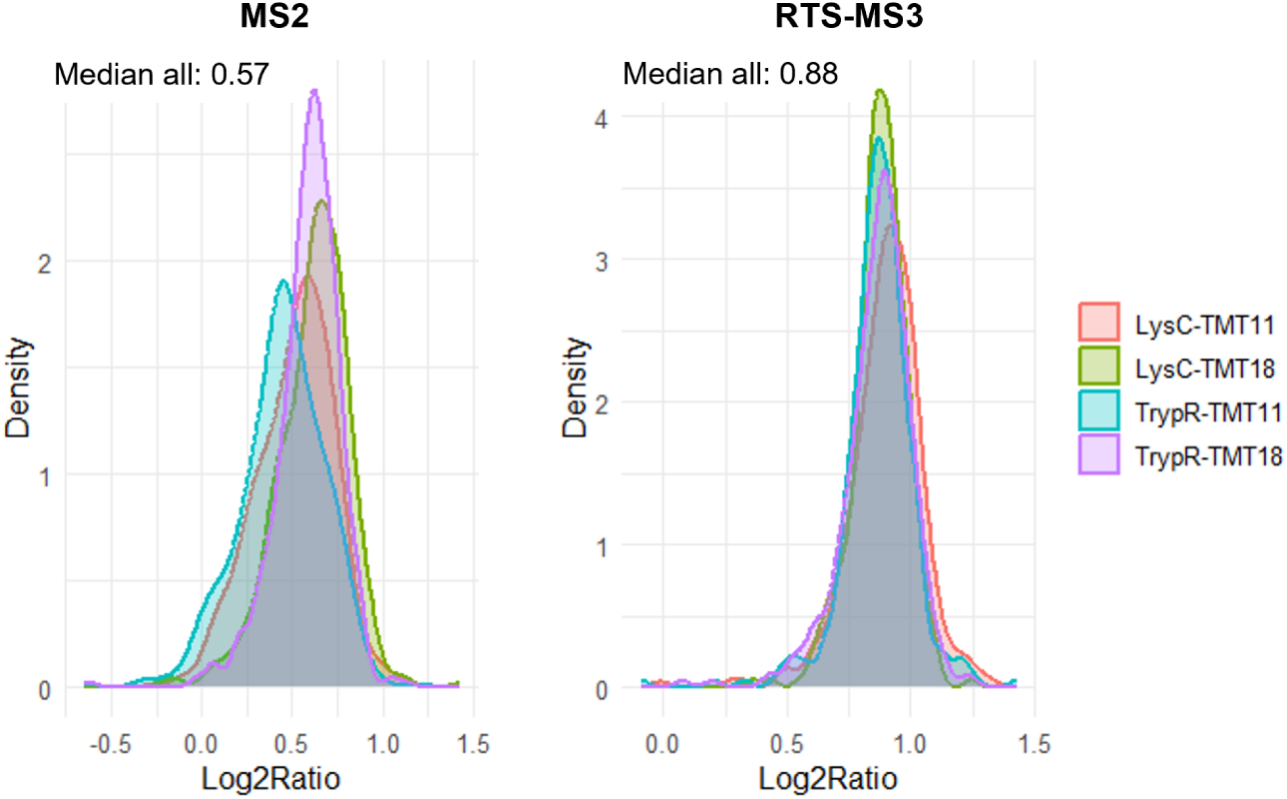
Quantification accuracy across UltraPlex-TMT subplexes using MS2 and RTS-MS3 acquisition. Density plots of log2 ratios for quantified *E. coli* proteins in a two-species benchmark system using MS2 (left) and RTS-MS3 (right) acquisition. Each distribution corresponds to a specific subplex combination of protease (LysC or TrypR) and TMT tag set (TMT11 or TMT18), as indicated by color.

## Conclusions

This study explored the design and performance of a streamlined and scalable proteomic workflow, UltraPlex-TMT, which combines orthogonal protease digestion with hyperplexed TMT and TMTpro labeling to significantly enhance sample pseudomultiplexing. By systematically evaluating peptide and protein-level outcomes across subplexes, we provide both practical benchmarking data and conceptual insights into the advantages and limitations of this strategy.

A key strength of UltraPlex-TMT lies in its practical simplicity; it is fully compatible with standard reagents, one-pot digestion to peptide labeling protocols, and widely available mass spectrometry instrumentation, requiring no specialized chemistry or hardware. By organizing samples in a 96-well plate into subplexes defined by enzyme specificity and labeling strategy, and recombining them postdigestion, UltraPlex-TMT effectively doubles sample capacity per experiment. This modular design not only improves throughput and efficiency but also facilitates robust statistical comparisons while remaining compatible with standard LC-MS setups. UltraPlex-TMT is well suited for high-throughput discovery (cell line panels, perturbations, time courses) and lower-complexity proteomes, where capacity and protein-level concordance are prioritized. Our approach extends existing methods via orthogonal protease cleavage, while reducing hands-on preparation and instrument time by fractionating once and acquiring subplexes together, making it resource-efficient for large studies.

Notably, the depth of protein quantification achieved across UltraPlex-TMT subplexes approached 7,000 proteins using standard MS2 acquisition. However, due to the distinct cleavage specificities employed, only ∼50% of all proteins were shared between subplexes, representing the fully overlapping, consistently quantified portion of the proteome. While this overlap is modest, it still reflects a substantial core proteome that is well-suited for robust comparative analyses and global biological interpretation. This overlap can be further increased by employing orthogonal cleavage as an alternative hyperplexing strategy on its own; however, this comes at the expense of reduced sample throughput. Although peptide and protein identifications were modestly higher in TMT18-labeled subplexes compared to their TMT11 counterparts, we interpret this less as a systematic advantage of TMTpro reagents and more as a consequence of increased cumulative peptide load across 18 channels. This suggests that further performance gains may be possible by scaling peptide input appropriately. As faster acquisition platforms such as the Orbitrap Astral^21^ become more widely adopted, the potential for deeper, more uniform coverage across complex hyperplexed designs is expected to increase by directly addressing the increased MS1 complexity through higher scan rates. In this context, UltraPlex-TMT may serve as a practical and scalable baseline strategy for high-throughput isobaric labeling-based proteomic studies. Moreover, UltraPlex-TMT is highly modular and could be extended through combination with other hyperplexing technologies. For instance, the integration of the recently reported 35-plex TMTpro reagents^3^ or peptide barcoding strategies like TAG-TMTpro^10^ could enable multiplexing on the order of 210 samples in a single experiment when paired with dual-protease UltraPlex-TMT designs. In the context of less complex proteomes, such as pull-down assays, subcellular fractions, or yeast lysates, where minimal or no fractionation is required, such an approach could support analysis throughput at rates below 5 min or even subminute per sample.

Quantitatively, the protein-level reproducibility and spike-in accuracy across subplexes support the robustness of the UltraPlex-TMT approach. The comparison between MS2 and RTS-MS3 acquisition modes revealed expected trade-offs: RTS-MS3 offered improved ratio accuracy and reduced interference, particularly for the *E. coli* spike-ins, but at the cost of lower protein quantification depth. Interestingly, this depth reduction was more substantial than would be predicted from peptide identification numbers alone. The partial mismatch between the real-time search engine and postacquisition database search could be responsible for missed MS3 triggers. The quantification depth of RTS-MS3 could potentially be improved by enabling the close-out option, which allows setting a cutoff on the maximum number of peptides to be triggered for MS3 per protein thereby addressing the increased MS1 complexity. However, our preliminary optimization experiments revealed a key limitation in this approach: when close-out was enabled, most of the TMT18-labeled peptides were not quantified. This was likely due to their more hydrophilic TMT11 counterparts eluting earlier and being prioritized for MS3 triggering, after which the TMT18 variants were excluded despite carrying distinct quantitative information. These results highlight a current shortcoming in standard peptide selection algorithms and point to the need for more intelligent acquisition logic tailored to complex hyperplexed designs such as UltraPlex-TMT. Furthermore, the quantification accuracy of MS2-based acquisition could be improved by implementing our previously described DIWA (Dual Isolation Width Acquisition) strategy,^22^ by utilizing high-field asymmetric-waveform ion mobility MS^23^ or by complement reporter ion quantification,^24^ which mitigate or eliminate interference effects and enhance quantitative accuracy on state-of-the-art instruments that lack MS3 capabilities.

Despite the inherent ratio compression associated with MS2 acquisition, particularly for reporter ions overlapping across all subplexes, the method still preserved biologically meaningful signals that closely mirrored those obtained via RTS-MS3. This was demonstrated through gene set enrichment analysis (GSEA) performed on the commonly quantified proteins, which revealed strong concordance in pathway-level outcomes between MS2 and MS3 data sets. Notably, across all comparisons and quantitative metrics, the use of orthogonal protease digestion did not introduce systematic biases. These findings support its value as a robust and unbiased multiplexing dimension that enhances TMT-based workflows without compromising data integrity.

In summary, UltraPlex-TMT provides a practical, flexible, and scalable foundation for high-throughput, isobaric labeling-based proteomics. Its performance is expected to benefit from advances in both emerging mass spectrometry hardware and more intelligent acquisition software. As multiplexing technologies continue to evolve, UltraPlex-TMT represents a robust platform that can be readily adapted and extended to meet the demands of large-scale proteomic studies.

## Supplementary Material





## Data Availability

The mass spectrometry proteomics data has been deposited to the ProteomeXchange Consortium via the PRIDE^25^ partner repository with the data set identifier PXD070352.

## Notes

Figure S1: proteolytic performance and TMT labeling efficiency in the UltraPlex-TMT workflow premix runs; Figure S2: peptide-level analytical characteristics across enzyme and TMT configurations; Figure S3: quantitative reproducibility and overlay of MS2 and RTS-MS3 data; Figure S4: distribution of relative protein abundance across TMT channels and experimental conditions for the *E. coli* spike-in proteins (PDF)
Table S1: unnormalized protein abundances (S/N) and log2 scaled values for each subplex for MS2 and RTS-MS3 acquisition (XLSX)

## Abbreviations

CID: Collision-Induced Dissociation
CV: Coefficient of Variation
DIWA: Dual Isolation Width Acquisition
GSEA: Gene Set Enrichment Analysis
HCD: Higher-Energy Collisional Dissociation
LC-MS/MS: Liquid Chromatography-Tandem Mass Spectrometry
LysC: Lysyl Endopeptidase
MAD: Median Absolute Deviation
MS: Mass Spectrometry
MS2: Tandem Mass Spectrometry, second stage (standard fragmentation)
MS3: Tandem Mass Spectrometry, third stage
PSM: Peptide Spectrum Match
PVCA: Principal Variance Component Analysis
RTS-MS3: Real-Time Search MS3
TMT: Tandem Mass Tag
TrypR: Trypsin with Specific Arginine Cleavage
