# Supplementary material for: UltraPlex-TMT: Expanding Isobaric Hyperplexing via Orthogonal Protease Cleavage
Source: J Proteome Res. 2026 Feb 2;25(3):1788–97. doi: 10.1021/acs.jproteome.5c01084 (PMC12973366; doi:10.1021/acs.jproteome.5c01084)
Supplement: Supplementary file 1 [file pr5c01084_si_001.pdf]

Supporting Information

UltraPlex-TMT: Expanding isobaric hyperplexing via orthogonal protease cleavage

Theodoros I. Roumeliotis[1,*], Fernando J. Sialana[1], Jenny Ho[2], Jyoti S. Choudhary[1,*]

[1]The Institute of Cancer Research, Chester Beatty Laboratories, London, SW3 6JB, UK

[2]Thermo Fisher Scientific, Stafford House, Hemel Hempstead, HP2 7GE, UK

[*]Correspondence should be addressed to J.S.C (jyoti.choudhary@icr.ac.uk) and T.I.R (theo.roumeliotis@icr.ac.uk)

Contents

Supplemental Figures S2

Figure S1. Proteolytic performance and TMT labeling efficiency in the UltraPlex-TMT workflow premix runs. S2

Figure S2. Peptide-level analytical characteristics across enzyme and TMT configurations. S3

Figure S3. Quantitative reproducibility and overlay of MS2 and RTS-MS3 data. S4

Figure S4. Distribution of relative protein abundance across TMT channels and experimental conditions for the E. coli spike-in proteins. S5

Supporting Tables S5

Table S1 (xlsx) S5

Supplemental Figures

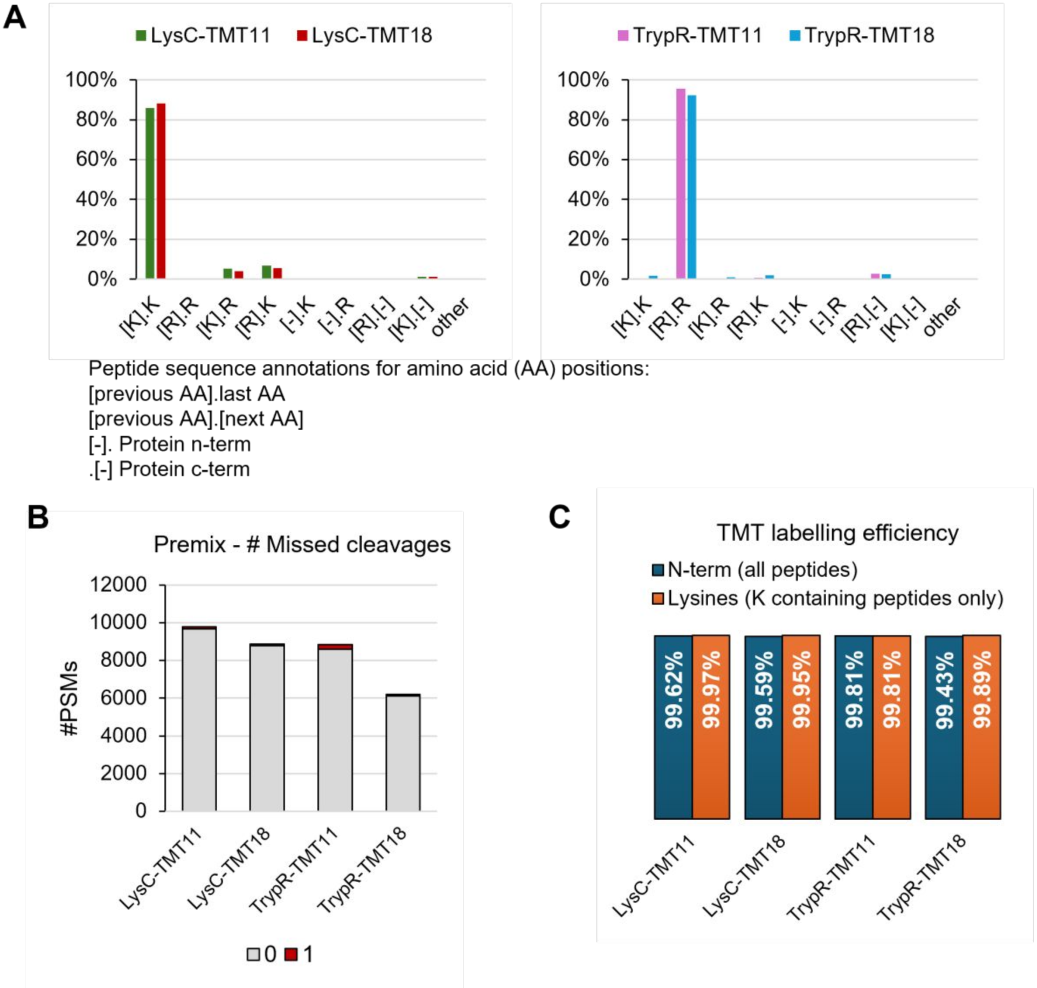

Figure S1. Proteolytic performance and TMT labeling efficiency in the UltraPlex-TMT workflow premix runs.

A) Cleavage patterns for each enzyme-TMT combination in the premix runs, analysed using the trypsin (cleavage at K and R) search setting.
B) Number of peptide-spectrum matches (PSMs) with 0 or 1 missed cleavage across different individual enzyme-TMT combinations using the LysC and TrypR -specific search settings.
C) Bar plots showing the N-terminal and lysine TMT labelling efficiency across all sub-plexes.

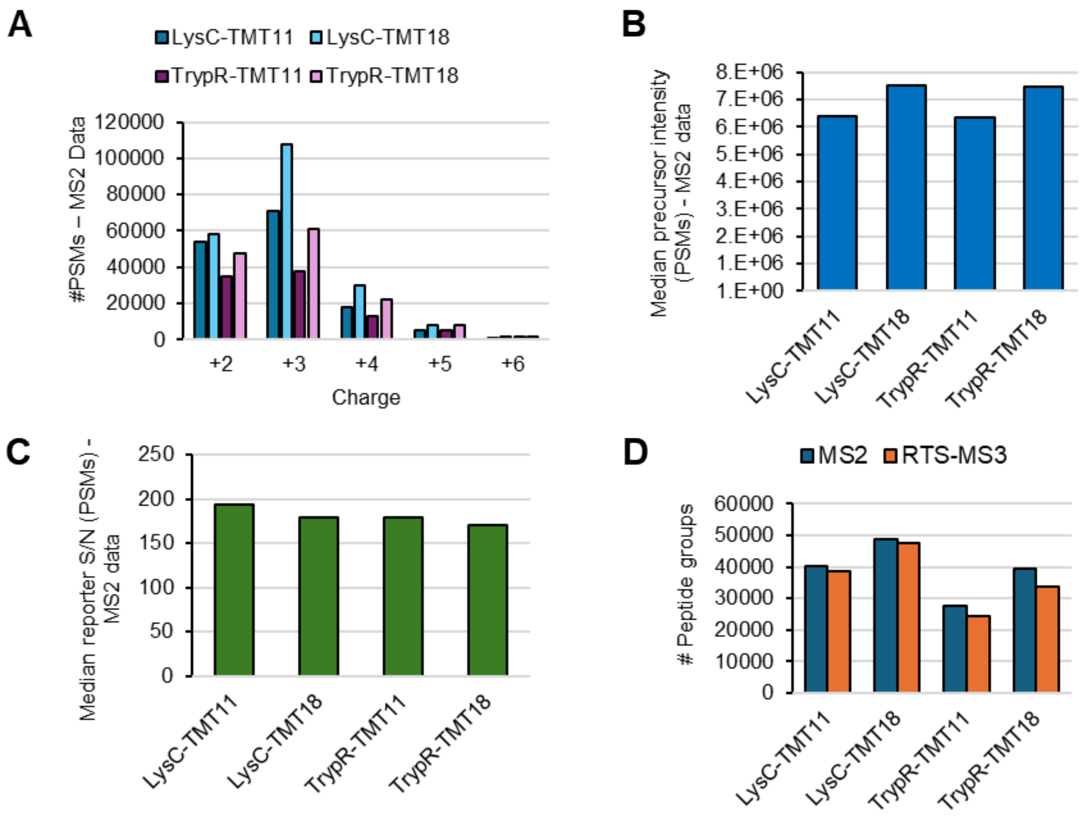

Figure S2. Peptide-level analytical characteristics across enzyme and TMT configurations.

A) Distribution of peptide-spectrum matches (PSMs) by precursor charge state for each enzyme-TMT combination in MS2 data.

B) Bar plots showing median precursor intensity across datasets.

C) Bar plots showing median reporter ion signal-to-noise (S/N) ratios for the MS2 data.

D) Bar plots showing number of quantified peptide groups identified using MS2 and RTS-MS3 acquisition modes.

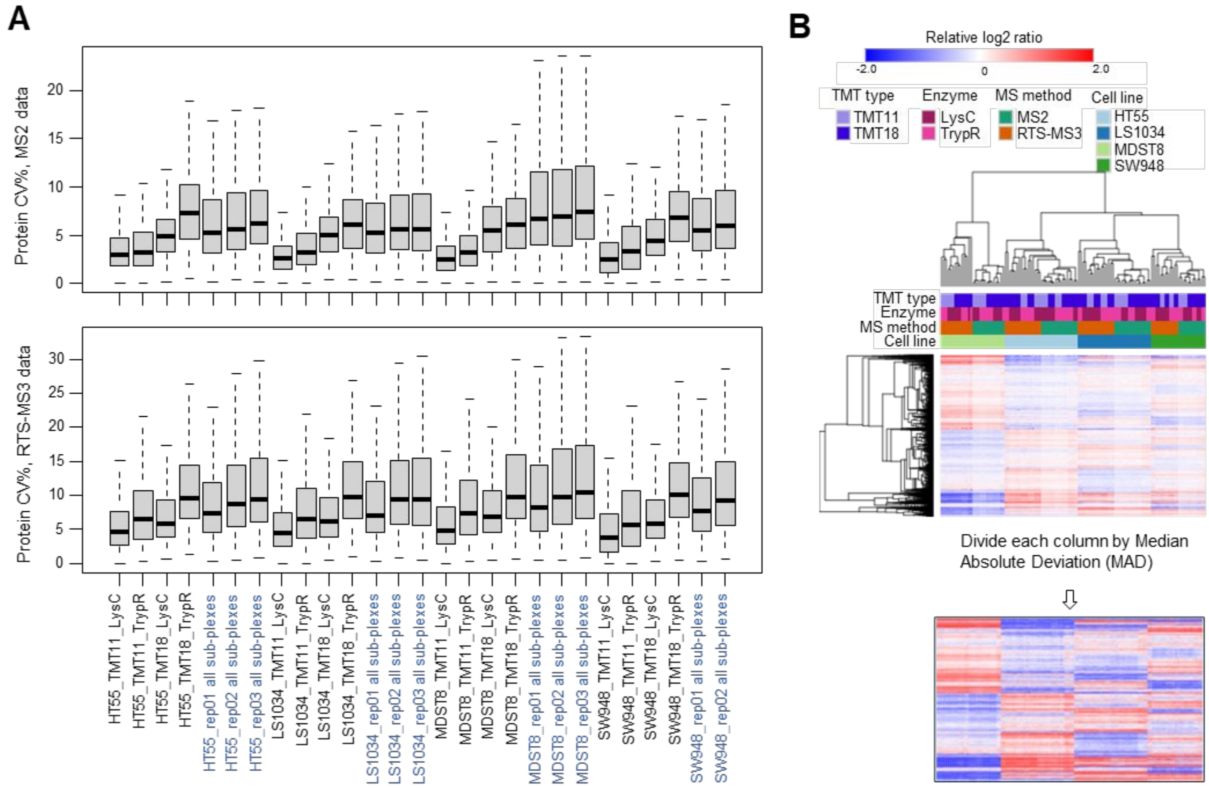

Figure S3. Quantitative reproducibility and overlay of MS2 and RTS-MS3 data.
A) Box plots showing protein coefficient of variation (CV%) between replicates within sub-plexes (black font) and between sub-plexes (blue font).
B) Hierarchical clustering of proteomic profiles from four colorectal cancer cell lines comparing different enzymes, TMT types, and MS acquisition methods, shown before and after median absolute deviation (MAD) normalization.

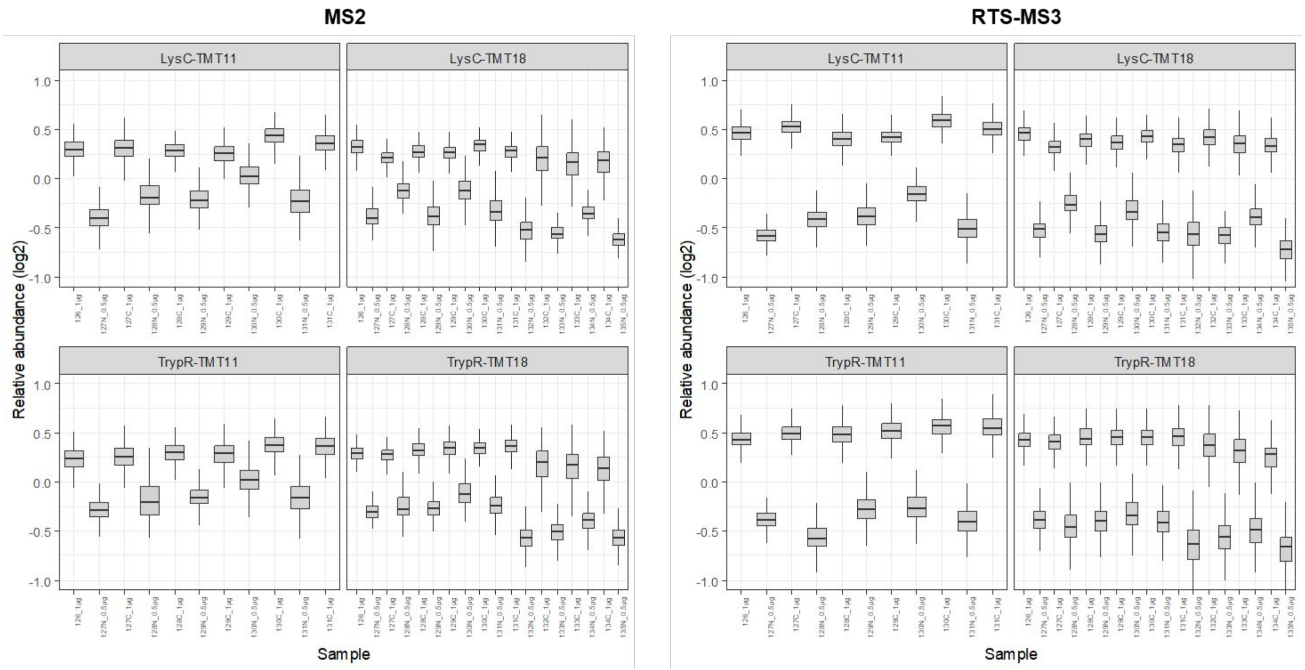

Figure S4. Distribution of relative protein abundance across TMT channels and experimental conditions for the E. coli spike-in proteins.

Boxplots represent log2-transformed relative abundance values per sample (TMT channel) for each sub-plex, stratified by enzyme (LysC or TrypsinR) and TMT labeling (TMT11 or TMT18) for MS2 (left panel) and RTS-MS3 (right panel) quantification.

Supporting Tables

Table S1 (xlsx). Unnormalized protein abundances (S/N) and log2 scaled values for each sub-plex for MS2 and RTS-MS3 acquisition.
